# 1,3-Dithiolane as a Privileged Scaffold in Bioactive Derivatives: Chiral Resolution and Assignment of Absolute Configuration

**DOI:** 10.3390/ijms252312880

**Published:** 2024-11-29

**Authors:** Roberta Listro, Giacomo Rossino, Valeria Cavalloro, Daniela Rossi, Massimo Boiocchi, Marina Simona Robescu, Teodora Bavaro, Silvia Franchini, Claudia Sorbi, Marco De Amici, Pasquale Linciano, Simona Collina

**Affiliations:** 1 Department of Drug Sciences, University of Pavia, Viale Taramelli 12, 27100 Pavia, Italy; roberta.listro@unipv.it (R.L.); giacomo.rossino@unipv.it (G.R.); daniela.rossi@unipv.it (D.R.); marinasimona.robescu@unipv.it (M.S.R.); teodora.bavaro@unipv.it (T.B.); pasquale.linciano@unipv.it (P.L.); 2 Department of Earth and Environmental Sciences, University of Pavia, Via Sant’Epifanio 14, 27100 Pavia, Italy; valeria.cavalloro@unipv.it; 3 Centro Grandi Strumenti, University of Pavia, Via Bassi 21, 27100 Pavia, Italy; massimo.boiocchi@unipv.it; 4 Department of Life Sciences, University of Modena and Reggio Emilia, Via Campi 103, 41125 Modena, Italy; silvia.franchini@unimore.it (S.F.); claudia.sorbi@unimore.it (C.S.); 5 Department of Pharmaceutical Sciences, University of Milan, Via Luigi Mangiagalli 25, 20133 Milan, Italy; marco.deamici@unimi.it

**Keywords:** 1,3-dithiolane, enantioselective chromatography, chiral resolution, X-ray diffraction, absolute configuration assignment

## Abstract

The 1,3-dithiolane ring has been recently rehabilitated as a chemical scaffold in drug design. However, for derivatives that are substituted in position 4, the introduction of a chiral center on the heterocycle demands the separation and characterization of the stereoisomers. We report the first chiral resolution and absolute configuration (AC) assignment for (1,4-dithiaspiro[4.5]decan-2-yl)methanol (*R/S*)-**1**, a key synthon for dithiolane-based biologically active compounds. Using (semi)preparative enantioselective HPLC, we isolated enantiomeric **1**. The AC was assigned by using (+)-**1** for the enantioselective synthesis of (+)-**BS148**, a sigma receptor modulator. An X-ray diffraction analysis established the (*R*)-configuration of (+)-**BS148** and, by extension, of (*+*)-**1**. This method provides a reliable approach for preparing enantiopure 1,3-dithiolane scaffolds and establishes reference standards for AC determination of related compounds.

## 1. Introduction

**Figure 1:**
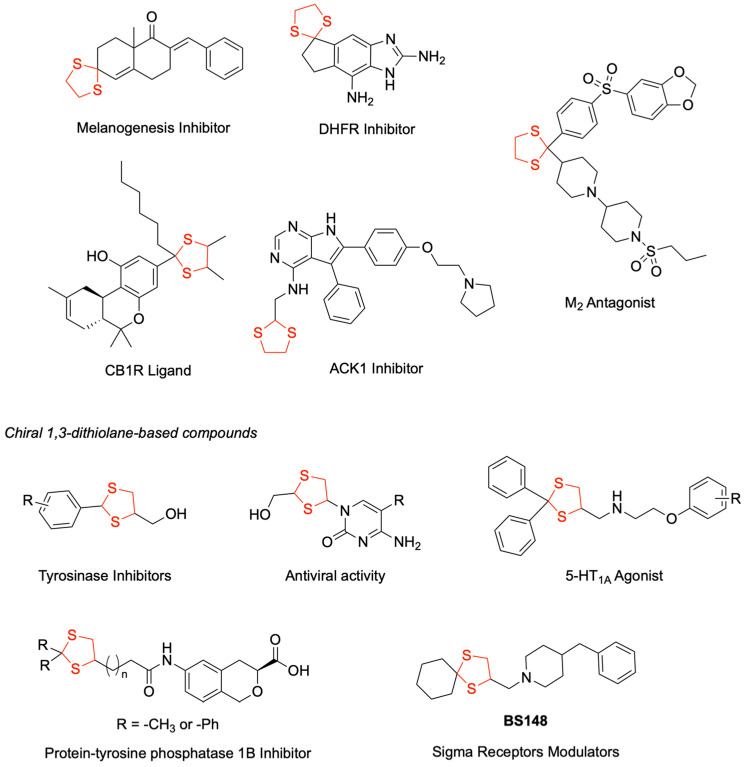
Examples of 1,3-dithiolane-containing biologically active compounds.

The 1,3-dithiolane heterocycle has been rehabilitated as a chemical scaffold in drug design, being incorporated into diverse therapeutic agents for treating different diseases, including neoplastic, infectious, inflammatory, and neurodegenerative disorders. As depicted in Figure 1, this scaffold has been the basis of many promising drug candidates. As an example, several M_2_ receptor antagonists with enhanced metabolic stability were reported [1]. Structure–activity relationship studies led to the identification of efficacious melanogenesis inhibitors [2]. Further medicinal chemistry efforts yielded cannabinoid CB1 receptor ligands possessing favorable drug-like properties and enhanced pharmacodynamic profiles [3]. The versatility of this scaffold is further demonstrated by its incorporation into dihydrofolate reductase (DHFR) inhibitors, which established a promising foundation for the development of novel anti-infective agents [4]. Of particular significance, a 1,3-dithiolane-functionalized 4,5,6-trisubstituted-7*H*-pyrrolo[2,3-d]pyrimidin-4-amine was identified as a potent ACK1 (Activated Cdc42-associated tyrosine Kinase 1) inhibitor, demonstrating excellent cellular activity, high kinase selectivity, and favorable in vitro metabolic profile. However, suboptimal pharmacokinetic parameters precluded its evaluation in tumor xenograft models [5].

The incorporation of the 1,3-dithiolane ring, particularly when substituted at position 4, might result in the introduction of a stereogenic center. Several bioactive compounds featuring chiral dithiolane moieties have demonstrated therapeutic potential (Figure 1). Notable examples include the series of (2-substituted phenyl-1,3-dithiolan-4-yl) methanols that were developed as tyrosinase inhibitors with antimelanogenic effects [6]. A series of isochroman mono-carboxylic acid derivatives were reported as protein tyrosine phosphatase 1B (PTP1B) inhibitors, where high potency was associated when a dithiolane ring was introduced as a side chain to the isochroman ring [7]. The therapeutic versatility of 1,3-dithiolane scaffolds was further exemplified by patented nucleoside analogs, which demonstrated promising antiviral activity [8].

A significant contribution was made by rational drug design when the bioisosteric replacement of 1,3-dithiolane in α1-adrenoceptor antagonists yielded selective 5-HT_1A_ agonists with enhanced potency. Extending this strategy, 1,4-dioxaspiro[4.5]decane derivatives were successfully transformed into cyclohexylspiro 1,3-dithiolane analogs, which exhibited nanomolar activity as sigma receptor modulators. Particularly noteworthy was compound **BS148** [9] (Figure 1), which showed selective toxicity against SK-MEL-2, a metastatic malignant melanoma cell line, while not affecting normal human melanocytes. Furthermore, in PDX models, **BS148** is effective in reducing cell viability and migration properties, showing potential as a future preclinical candidate for treating metastatic melanoma [10].

Despite the relevance of the 1,3-dithiolane moiety in medicinal chemistry—and the known concept that stereochemistry is an essential feature of biologically active compounds, since it can influence the pharmacodynamic, as well as the pharmacokinetic, properties and impact the toxicology [11,12]—the enantiomeric resolution of compounds possessing a stereocenter on this heterocycle has been poorly investigated [13,14]. At present, these chiral 1,3-dithiolanes have been synthesized and biologically evaluated as racemates.

**Figure 2:**
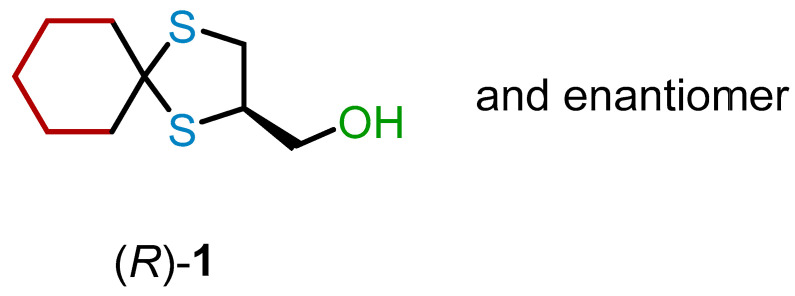
Structure of 1,3-dithiolane (*R*)-(1,4-dithiaspiro[4.5]decan-2-yl)methanol **1**.

In this work, motivated by our interest in 1,3-dithiolane **1** (Figure 2), we systematically investigated various approaches for its chiral resolution and the assignment of the absolute configuration (AC) to the enantiomers.

## 2. Results and Discussion

**Scheme 1:**
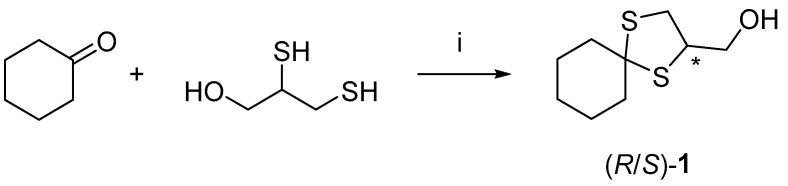
Reagents and conditions: (i) cyclohexanone (1 equiv.), 2,3-dimercaptopropan-1-ol (1 equiv.), HClO_4_/SiO_2_ (20 mg per mmol), neat, r. t., 1 h, 30% yield. The chiral center of product **1** is indicated with an asterisk (*).

Alcohol (±)-**1** was synthesized on a gram scale using the procedure already published by Franchini et al. [9] (Scheme 1).

Initially, an attempt at chiral resolution of compound **1** was performed via diastereomeric esters, a strategy that is still widely employed in industrial settings due to its reliability and simplicity [15]. Diastereomeric esters were synthetized, using enantiopure carboxylic acids or sulfonyl chlorides (i.e., Fmoc-*L*-phenylalanine, Fmoc-*R*-tyrosine, (*S*)-(+)-10-camphorsulfonyl chloride, and (1*R*)-(+)-3,4 dimethyl aryloxypropionic acid). Different solvent systems were systematically evaluated to achieve separation of the resulting diastereomers based on their solubility differences. However, the recovery was poor and insufficient for separation on a hundred-milligram scale. Therefore, RP-HPLC was investigated as an alternative approach for the separation of the diastereomeric esters of **1** on a (semi)preparative scale, followed by hydrolysis to recover enantiomeric **1**. Despite these efforts, enantiomeric **1** was not isolated in a suitable amount for further studies, leading to the discontinuation of this approach.

**Scheme 2:**
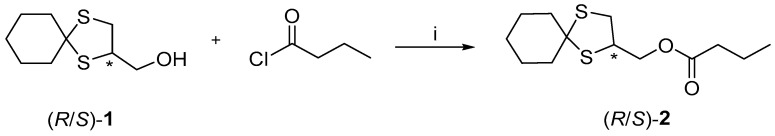
Reagents and conditions: (i) **1** (1 equiv.), butyryl chloride (1 equiv.), 4-dimethylaminopyridine (cat), anh. DCM, nitrogen atmosphere, 0 °C to r.t., 7 h, 95% yield. The chiral centers on the substrate and on the product are indicated with an asterisk (*).

A biocatalytic approach was therefore investigated as a subsequent attempt to identify an efficient and easy-to-scale chiral resolution method. In fact, biocatalysis is universally recognized as a powerful tool for the efficient preparation of homochiral drugs [16]. Based on our previous experience, alcohol (*R/S*)-**1** was converted to the butyryl ester (*R*/*S*)-**2** (Scheme 2), as esterification provides a substrate suitable for enzymatic kinetic resolution, where hydrolytic enzymes can selectively cleave one enantiomer of the ester while leaving the other unchanged, thereby enabling the separation of the two enantiomers through different reaction rates [17].

To monitor the enzymatic hydrolysis and assess the enantiomeric purity of product **1**, an analytical chromatographic resolution method on Chiral Stationary Phases (CSPs) was developed.

**Figure 3:**
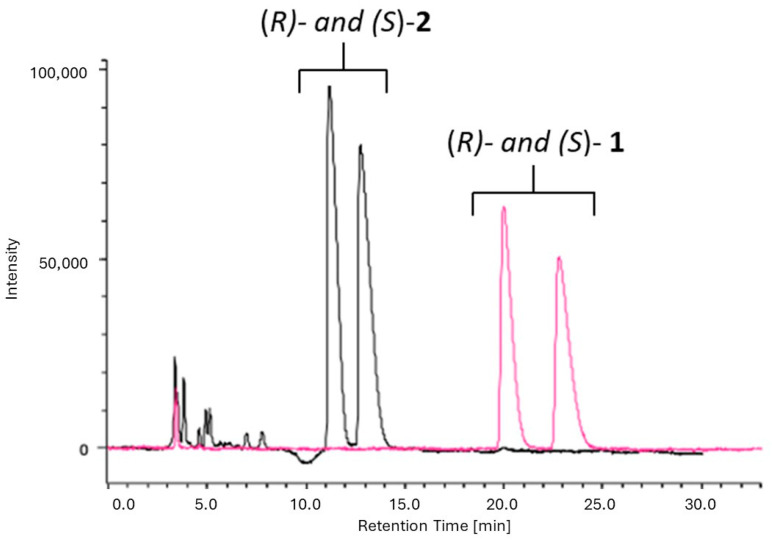
UV/vis-HPLC trace (λ = 220 nm) for compounds (*R/S*)-**2** and (*R/S*)-**1** on Chiralpak IA (4.6 × 250 mm, 5 μm); eluent *n*-Hex/MTBE 4:1 (*v*/*v*); flow rate: 1 mL/min; 25 °C. Injection volume: 10 μL; concentration: 1 mg/mL.

A comparison was performed between two chiral selectors based on polysaccharides consisting of amylose- and cellulose-derived CSPs. Based on our previous experience [18,19] Chiralpack IA (amylose-based CSP, 0.46 cm diameter × 25 cm length, 5 μm) and Chiralpak IC (cellulose-based CSP, 0.46 cm diameter × 25 cm length, 5 μm) columns were selected for this study. HPLC screening was performed under two possible chiral separation modes, namely, normal and reverse-phase. Accordingly, the mobile phases investigated were a mixture of *n*-hexane (*n*-Hex) and 2-propanol (IPA) or ethanol or methyl tert-butyl ether (MTBE) as polar modifiers, or pure alcohols (IPA or ethanol). Several types of mobile phase compositions were investigated by changing the percentages of the mixture components. The main results of the several assessed protocols, expressed in terms of the retention factors (*k_1_* and *k_2_*), separation factor (α), and resolution factor (*Rs*), are reported in Table S4. Notably, the best chromatographic conditions for separation of the enantiomers of alcohol **1** (see Table S4, conditions E and H) were also tested for ester **2**. Only condition H allowed for the resolution of ester **2**, thus enabling the monitoring of the enzymatic reaction progression in one single run. According to the screening results, suitable baseline separation of both alcohol **1** (*k*_1_ = 4.60; *k*_2_ = 5.34; α = 1.16; *Rs* = 1.39) and ester **2** (*k*_1_ = 2.18; *k*_2_ = 2.63; α = 1.21; *Rs* = 1.60) was obtained on Chiralpak IA with *n*-Hex:MTBE 4:1 *v*/*v* at a flow rate of 1 mL/min (Figure 3).

With the analytical method being established, a set of hydrolytic enzymes, including lipases, esterases, proteases, and acylases, that were previously immobilized in-house (Figures S1–S3 and Tables S1–S3) on various carriers (except Novozyme435^®^, which is commercially available) (Table 1) were tested [20,21]. The initial screening was performed using 10 mM of (*R/S*)-**2** in *tert*-butanol and water (90:10 *v*/*v*), and 68 units of enzyme *per* mg of substrate were incubating at room temperature on a rolling shaker (Scheme S1). The progression of the reaction was monitored by HPLC analysis at two time points (6 and 24 h) to determine the degree of conversion (ester–alcohol) and the enantiomeric excess (*ee*) of both ester **2** and alcohol **1**. Experimental details about the enzyme immobilization, activity determination, biocatalyzed reaction, and monitoring are reported in the Supplementary Materials.

All the enzymes tested were able to hydrolyze the substrate (*R/S*)-**2** with good conversion rates (>60%). Despite the good conversion, all the lipases tested completely lacked enantioselectivity. Only acylase and ANE yielded the target alcohol **1** with nearly full conversion, although with low enantioselectivity (*ee* of 25% and 30% after 24 h, respectively). Subsequent optimization attempts were performed with both acylase and ANE, monitoring the reaction before the 24 h end point (2 h, 4 h, 7 h, 10 h, 13 h, 16 h, 19 h, and 24 h). However, these experiments did not lead to a significant improvement in enantioselectivity (Figures S4 and S5). As a result, these data limit the applicability of the enzymatic approach on a preparative scale.

**Figure 4:**
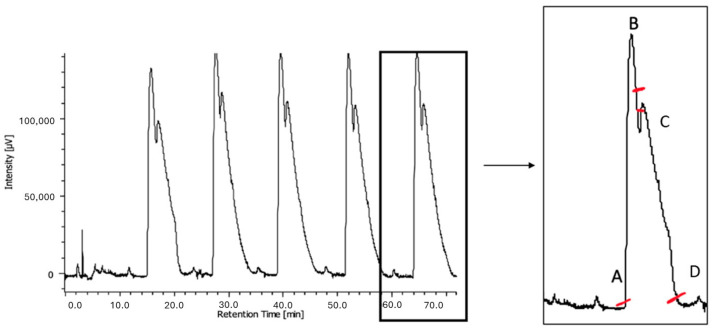
UV/vis-HPLC trace (λ = 230 nm) for (semi)preparative separation of compound (*R/S*)-**1**. Chiralpak IA (250 mm × 1 cm, 5 μm), *n*-Hex/MTBE 4:1 (*v*/*v*), flow rate of 5 mL/min, 25 °C. Injection volume: 100 μL; concentration: 30 mg/mL. According to the UV profile, the fractionation was performed following the dashes (first eluted from A to B, mixed fractions from B to C, and second eluted from C to D).

We therefore attempted the resolution of (*R*/*S*)-**1** via (semi)preparative chiral chromatography separation, scaling up the above-described analytical protocol. The chromatographic resolution was carried out on a Chiralpak IA column (250 mm × 1 cm, 5 µm), using a mixture of *n*-Hex and MTBE (4:1, *v*/*v*) at a 5 mL/min flow rate. This method has an essential feature for economic and productive preparative enantiomer separation, i.e., the high solubility of the racemate and enantiomers in the eluent/injection solvent. Conversely, the composition of the mobile phase is a weakness, as it cannot be reused. To overcome the limitation related to the non-recyclable nature of the mobile phase and to improve the environmental sustainability of the protocol, continuous injections of the racemate were carried out. Moreover, injection parameters, such as sample concentrations (10, 20, and 30 mg/mL) and injection volumes (100 and 250 μL), were optimized to achieve a proper balance between the amount of sample injected and the chromatographic resolution. Optimal separation conditions were achieved with cycles of 11 min of continuous sample injections (100 µL) at a 30 mg/mL concentration (Figure 4).

Through this optimized protocol, 100 mg of racemic **1** was processed in three cycles of injections. The eluate was properly partitioned according to the UV profile (Figure 4, right), leading to the isolation of 34 mg of the first eluted enantiomer with an *ee* of 99.9% (${\left[\alpha \right]}_{D}^{30}$ = −20.5, c ≅ 0.5%, DCM) and 43 mg of the second eluted enantiomer with an *ee* of 85.1%. A subsequent purification step of the second fraction (five injections) led to the isolation of 32 mg of the second eluted enantiomer (recovery 64%) with an *ee* of 95.6% (${\left[\alpha \right]}_{D}^{30}$= +17.9; c ≅ 0.5%; DCM).

Summarizing, using the conditions described above, the separation of 100 mg of (*R*/*S*)-**1** was accomplished within one working day, consuming approximately 3 L of mobile phase.

The quantity of isolated enantiomers of **1** was sufficient for AC assignment studies. Among the experimental methods available, electronic and vibrational circular dichroism (ECD and VCD, respectively) were not applicable due to the lack of reference compounds in the literature to which the VCD or ECD spectra could be compared. Therefore, we opted for X-ray diffraction, which is the gold-standard technique for AC assignment, due to its reliability of data interpretation. Despite different crystallization trials with (−)-**1** being performed using various solvents and techniques such as slow cooling, evaporation, steam, and vapor diffusion [22], all attempts failed, probably owing to the flexibility of the molecule.

**Scheme 3:**
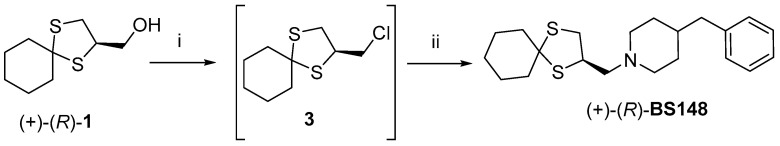
Reagents and conditions: (i) thionyl chloride (2 equiv.), DMF (cat.), anh. DCM, r.t., 15 min; (ii) 4-benzylpiperidine (2 equiv.), KI (cat), DMSO, 90 °C, 18 h.

For this reason, we shifted our attention to **BS148** (Figure 1), which possesses the same spyro cyclohexyldithiolane scaffold and is prepared from **1**, based on the hypothesis that it might be more easily crystallized from its precursor owing to its structural features [9]. Accordingly, (+)-**1** was used for the enantioselective synthesis of the corresponding homochiral **BS148**. Briefly, as reported in Scheme 3, (+)-**1** was converted first into the alkyl chloride **3** by reaction with thionyl chloride in anhydrous DCM at room temperature for 15 min, using DMF as a catalyst. Intermediate **3** was reacted with 4-benzylpiperidine under modified S_N_2 conditions (the inorganic base was avoided to prevent and/or limit racemization) to achieve **BS148**.

**Figure 5:**
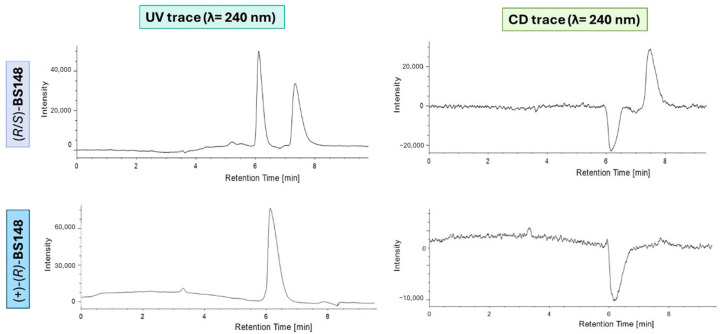
UV/vis-HPLC trace (λ = 240 nm) for analytical separation of **BS148**. Chiralpak IA (4.6 mm × 1 cm, 5 μm), *n*-Hex/MTBE/DEA 85:15:0.1. Flow rate:1 mL/min; 25 °C; injection volume: 10 μL. UV/vis-HPLC and ECD-HPLC trace with λ = 240 nm. (*R*/*S*)-**BS148**: *k*_1_ = 0.68; *k*_2_ = 1.00; α = 1.42; *R*_S_ = 2.20. Chromatogram and ECD trace of (*R*/*S*)-**BS148** are reported on the top, and chromatogram and ECD trace of (+)-(*R*)-**BS148** are reported on the bottom.

To verify whether racemization occurred throughout the synthetic procedure, a HPLC analytical resolution method for **BS148** was set up by making a slight modification to the mobile phase composition that was used for the separation of (*R/S*)-**1**. The chromatographic eluting conditions, together with the UV and ECD traces, are reported in Figure 5. The use of the chiroptical detector allowed us to simultaneously determine the enantiomer elution order at the analytical stage.

The analysis showed that the thus obtained **BS148** has an *ee* value of 99.9% (${\left[\alpha \right]}_{435}^{30}$ = +24.8, c ≅ 0.3%, DCM), meaning that, as foreseen, no evidence of racemization in the synthetic steps occurred.

The enantiopure sample of (+)-**BS148** was therefore amenable and subjected to crystallization studies.

**Figure 6:**
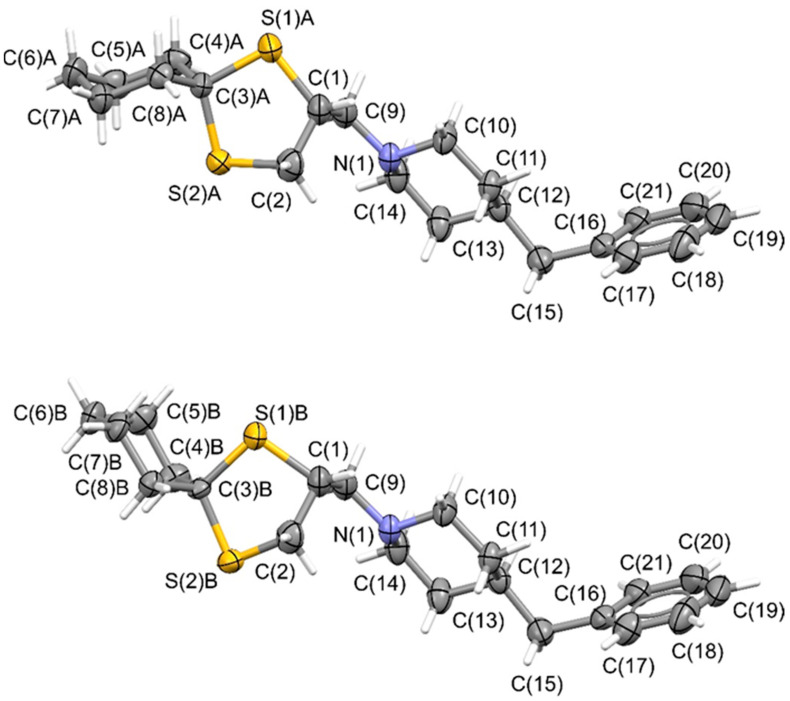
Plots showing thermal ellipsoids for the two (+)-**BS148** molecular constituents of the crystal (ellipsoids for non-H atoms are drawn at the 30% probability level). Due to the positional disorder affecting the molecules in the crystal, two alternative conformations of the 1,3-dithiolane ring and of the terminal cyclohexane moiety are present.

A high-quality needle-shaped single crystal of (+)-**BS148** was successfully obtained through slow-evaporation crystallization from ethanol at room temperature, and a crystallographic analysis was then performed. The crystal structure was solved in the Sohncke *P*2_1_2_1_2_1_ space groups (crystal data are reported in Table S5) and showed positional disordering resulting from the presence of two mutually exclusive conformations of the cyclohexylspyrodithiolane scaffold (top and bottom of Figure 6). In these two conformations, the 1,3-dithiolane rings exhibited slightly different spatial orientations, while the cyclohexyl ring was arranged in chair conformations as an almost regular envelope (top of Figure 6) or in a slightly twisted shape (bottom of Figure 6). Nevertheless, the chiral C(1) atom was not affected by this positional disorder. To assess the correctness of the determined absolute structure, and therefore the absolute configuration of the chiral C(1) atoms, we investigated the anomalous scattering effects in the measured reflections by analyzing the value of the *x* Flack parameter and of its standard uncertainty *u*. Based on the anomalous scattering effects [23], the final *x*(*u*) Flack parameter was −0.01(7). The *x* value of −0.01 was near to 0 (i.e., the value for a correctly assigned absolute structure) but was outside its physically meaningful range (from 0 to 1). Moreover, the *u* value of 0.07 was in the range of 0.04–0.10, for which validation of the enantiopurity of the starting material has to be assessed prior to the assignation of the absolute configuration of an analyte [24]. In our case, the *u* value was acceptable for the absolute configuration assignment, since we demonstrated the enantiopurity of the compound that was subjected to the crystallographic studies (*ee* > 99.9%, as reported in Figure 5) [25,26,27].

Accordingly, the combination of the X-ray crystallography and the prior determination and chiroptical characterization by CSP HPLC and in-line CD spectra of the enantiopurity of (+)-**BS148** allowed us to unequivocally establish the (*R*)-configuration for (+)-**BS148** and therefore for the key precursor (+)-**1**.

Summarizing, we developed a (semi)preparative method that is suitable for achieving quick access to the desired enantiomers with high enantiomeric excess and sufficient amounts for subsequent AC studies. Thanks to the high-concentration continuous injections, this approach is efficient in terms of time and solvent consumption. This method may be further developed for production of gram to Kg amounts of pure enantiomers that are needed for studying the role of chirality in biologically active compounds. Moreover, the X-ray analysis data, together with the prior characterization of the enantiopurity of the synthesized (+)-**BS148**, allowed for the indirect assignment of the AC to its precursor (+)-(*R*)-**1** and to the corresponding enantiomer (−)-(*S*)-**1**.

## 3. Materials and Methods

### 3.1. General Information

All commercially available reagents and solvents (Merck, Milan, Italy) were reagent-grade and were used without further purification, unless otherwise specified. Air/water-sensitive reactions were performed using oven-dried glassware and under a nitrogen atmosphere. The following solvents and reagents have been abbreviated: ethyl ether (Et_2_O), dimethyl sulfoxide (DMSO), ethyl acetate (EtOAc), dichloromethane (DCM), ethanol (EtOH), dimethylformamide (DMF), 4-dimethylaminopyridine (DMAP), *n*-hexane (*n*-Hex), *n*-propanol (IPA), and methyl tert-butyl ether (MTBE). All the compounds and intermediates were purified by flash chromatography on a silica gel stationary phase (60 Å; 230–400 Mesh). Reactions were monitored by thin-layer chromatography (TLC) on silica gel pre-coated glass-backed plates (60-F254; Merk) and visualized using UV light (λ = 254 and 366 nm, MinUVIS DESAGA^®^ Sarstedt-GRUPPE, Wiesloch, Germany), ninhydrin, or a cerium ammonium sulfate molybdate solution. NMR spectra were recorded on a Bruker Avance 400 spectrometer equipped with a BBI 5 mm probe, with ^1^H at 400.134 MHz and ^13^C at 100.62 MHz. Spectra were processed with the Top Spin software 4.4.0 from Bruker. Proton and carbon chemical shifts were referenced to the residual solvent peak. Chemical shifts are expressed in parts per million (ppm or δ). Coupling constants are reported in Hz. Multiplicity patterns are reported as follows: s (singlet), d (doublet), t (triplet), q (quartet), m (multiplet), and brs (broad signal). Enzymatic activity assays were monitored by Titrator 718 stat (pH-Stat) Tritino from Metrohm (Herisau, Switzerland). HPLC-UV/Vis analysis was carried out on a JASCO (Tokyo, Japan) system composed of a PU-2089 pump, AS-2055 autosampler, CO-4065 column oven, MD-1510 photodiode array (PDA) detector, and CD-2095 Electronic Circular Dichroism (ECD) Plus detector. The (semi)preparative chromatographic resolutions were performed on a Jasco system (JASCO Europe, Cremella, LC, Italy) equipped with a PU-2089 plus quaternary gradient pump, AS-2055 plus autosampler, MD-1510 Photo Diode Array (PDA) detector, and Electronic Circular Dichroism (ECD) 2095 Plus detector. Chromatograms were acquired and processed using the ChromNAV v 2.0 software (Tokyo, Japan). Optical rotation values were measured on a JASCO photoelectric polarimeter DIP 1000 with a 0.5 dm quartz cell at the sodium D line (λ = 589 nm); compounds were dissolved in DCM at a concentration of 0.5% (*w*/*v*), unless otherwise stated. X-ray diffraction data were collected on a Enraf-Nonius CAD4 four-circle diffractometer (Enraf-Nonius, Delft, The Netherlands) at room temperature with graphite monochromated Mo-Kα X-radiation (λ = 0.7107 Å).

### 3.2. HPLC Analysis

Enantiomeric resolutions for (*R/S*)-**1**, (*R/S*)-**2**, (*R/S*)-**BS148**, and (+)-(*R*)-**BS148** were achieved as follows:

*Method A* was adopted for (*R/S*)-**1** and (*R/S*)-**2.** Chiralpak IA column (4.6 × 250 mm, 5 µm) was used with *n*-Hex/MTBE 4:1 (*v*/*v*) as the mobile phase, with a flow rate of 1 mL/min, a sample concentration of 1 mg/mL, an injection volume of 10 μL, and UV/vis-HPLC trace (λ = 240 nm). The elution allowed us to obtain a baseline separation of both compounds. Results are expressed as retention factors (*k*_1_ and *k*_2_), separation (α), and resolution (*R_S_*) factors. Specifically, (*R/S*)-**1** showed k_1_ = 4.60; k_2_ = 5.34; α = 1.16; and *Rs* = 1.39. The ester (*R/S*)-**2** showed *k*_1_ = 2.18; *k*_2_ = 2.63; α= 1.21; and *Rs* = 1.60.

*Method B* was adopted for **BS148.** A Chiralpak IA column (4.6 × 250 mm, 5 µm) was used with *n*-Hex/MTBE/Diethylamine 85:15:0.1 (*v*/*v*/*v*) as the mobile phase, with a flow rate of 1 mL/min, a sample concentration of 1 mg/mL, an injection volume of 10 μL, UV/vis-HPLC trace (λ = 240 nm), and ECD-HPLC trace (λ = 240 nm). The elution allowed us to obtain a baseline separation. Results are expressed as retention factors (*k*_1_ and *k*_2_), separation (α), and resolution (*R_S_*) factors. Specifically, (*R/S*)-**BS148** showed *k*_1_ = 0.68; *k*_2_ = 1.00; α = 1.42; and *R*_S_ = 2.20. (+)-(*R*)-**BS148** showed *k*_1_ = 0.68.

### 3.3. Synthesis of (1,4-Dithiaspiro[4.5]decan-2-yl)methanol (R/S)-1

Under a nitrogen atmosphere and at room temperature, 2,3-dimercaptopropan-1-ol (1.26 g, 10.19 mmol, 1 equiv.) and HClO_4_ absorbed on SiO_2_ (70% *v*/*w*) (20 mg *per* mmol) were added to cyclohexanone (1 g, 10.19 mmol, 1 equiv.) The suspension was reacted neat under the same conditions for 1 h and quenched by the addition of EtOAc. The suspension was filtered and concentrated. The crude product was directly purified over a silica gel (ratio crude/silica 1:100, eluent DCM 100%) to obtain the desired product (310 mg, 30.5% yield) as a whitish oil. The ^1^H and ^13^C spectra of the racemate are in accordance with the literature [17].

### 3.4. Synthesis of (1,4-Dithiaspiro[4.5]decan-2-yl)methyl Butyrate, (R/S)-2

(±)-**1** (250 mg, 1.22 mmol, 1 equiv.) was dissolved in 7 mL of anhydrous DCM at room temperature and under a nitrogen atmosphere, followed by the addition of butyryl chloride (252 μL, 1.22 mmol, 1 equiv.), triethylamine (278.3 μL, 2.447 mmol, 2 equiv.), and a catalytic amount of DMAP. After 8 h, the reaction was stopped. The mixture was concentrated under reduced pressure. The crude product was resuspended in Et_2_O, and the organic phase was washed with 10% *v*/*v* HCl and a saturated solution of NaHCO_3_. The organic phase was dried over anhydrous Na_2_SO_4_ and concentrated to obtain 315 mg (95% yield) of the titled compound as a white oil. ^1^H NMR (400 MHz, CDCl_3_): δ 4.18–4.11 (dd, 1H), 4.01–4.04 (dd, 1H), 3.89–3.81 (m, 1H), 3.29–3.22 (dd, 1H), 3.17–3.11 (dd, 1H), 2.26–2.19 (t, 2H), 1.97–1.87 (dt, 4H), 1.66–1.48 (m, 6H), 1.37–1.30 (m, 2H), and 0.92–0.85 (t, 3H).

### 3.5. (Semi)preparative HPLC Separation

A Hamilton (Reno, NV, USA) syringe (syringe volume: 200 µL; loop: 200 µL) was employed for the (semi)preparative chiral resolutions. (Semi)preparative enantiomer (*R/S*)-**1** separation was performed on a Chiralpak IA (250 mm × 1 cm, 5 µm), using *n*-Hex/MTBE 4:1 (*v*/*v*) as mobile phase with a flow rate of 5 mL/min, a sample concentration of 30 mg/mL, and an injection volume of 100 µL.

The processing of 100 mg of racemic **1** resulted in 34.1 mg of the first eluted enantiomer (*ee =* 99.95%, recovery 68%) and 43.4 mg of the second eluted enantiomer (*ee* of 85.1%), together with 17.4 mg of an intermediate fraction as a mixture of the two isomers. A subsequent purification step of the second fraction led to the isolation of 32.1 mg of the second eluted enantiomer (*ee* of 95.60%, recovery 64%). The chiroptical properties of the enantiomers are reported as follows: (−)-(*S*)-**1**: ${\left[\alpha \right]}_{D}^{30}$ = −20.5 (c ≅ 0.5%, DCM), *ee =* 99.95%; (+)-(*R*)-**1**: ${\left[\alpha \right]}_{D}^{30}$ = + 17.9 (c ≅ 0.5%, DCM), *ee =* 95.60%.

### 3.6. Synthesis of 1-((1,4-Dithiaspiro[4.5]decan-2-yl)methyl)-4-benzylpiperidine (+)-(R)-BS148

To a solution of (±)-**1** (34 mg, 0.16 mmol, 1 equiv.) in anhydrous DCM, at room temperature and under a nitrogen atmosphere, thionyl chloride (21.8 µL, 0.32 mmol, 2 equiv.) and a catalytic amount of DMF were added, and the mixture was stirred under the same conditions for 15 min. The reaction was quenched by the addition of Et_2_O, and the organic phase was washed with a saturated solution of NaHCO_3_ and brine. The organic phase was dried over anhydrous Na_2_SO_4_ and concentrated to obtain the corresponding alkyl chloride **3** (33.5 mg, 94% yield), which was directly used in the next step without further purification.

Chloride **3** (33.5 mg, 0.15 mmol, 1 equiv.) was solubilized in DMSO (2 mL), and 4-benzyl piperidine (52.7 mg, 0.30 mmol, 2 equiv.) and KI (cat.) were added. The mixture was reacted at 90 °C overnight. Thereafter, the reaction was quenched with a saturated solution of Na_2_CO_3_, and the aqueous phase was extracted with EtOAc. The organic phase was washed with brine, dried over anhydrous Na_2_SO_4_, and concentrated. The crude was purified over a silica gel (ratio crude/silica 1:100, eluent *n*-Hex/AcOEt 8:2) to obtain 37.9 mg (70% yield) of the titled compound as a pale-yellow oil. TLC Rf: 0.37 (eluent *n*-Hex/AcOEt 8:2).

^1^H NMR (400 MHz, Chloroform-d) δ 1.30–1.67 (m, 11H), 1.91–2.11 (m, 6H), 2.46 (dd, *J* = 6.4, 12.8 Hz, 1H), 2.58 (d, *J* = 6.8 Hz, 2H), 2.71 (dd, *J* = 8.4, 12.4 Hz, 1H), 2.89–2.97 (m, 2H), 3.24 (dd, *J* = 6.4, 12.0 Hz, 1H), 3.33 (dd, *J* = 4.8, 6.8 Hz, 1H), 3.91–3.94 (m, 1H), 7.18–7.35 (m, 5H). (+)-(*R*)*-***BS148**: (${\left[\alpha \right]}_{435}^{30}$ = +24.8, c ≅ 0.3%, DCM), and *ee =* 99.9%.

### 3.7. X-Ray Crystallography Diffraction Analysis

A single crystal of (+)-**BS148** was obtained through slow-evaporation crystallization from ethanol at room temperature. Data reduction (including intensity integration, background, and Lorentz and polarization corrections) was performed with the WinGX software package—version 2023 [28], and absorption effects were evaluated by using the psi-scan semi-empirical method [29]. The absorption effects were found to be negligible, and absorption correction was not applied to the data. The crystal structure was solved by direct methods (SIR 97) [30] and refined by full-matrix least-square procedures on *F*^2^ using all reflections (SHELXL-2019/3) [31]. All non-H atoms were refined anisotropically, and hydrogen atoms were placed at calculated positions with the appropriate AFIX instructions and refined using a riding model. Positional disorder affected the 1,3-dithiolane ring and the terminal cyclohexane ring, which adopted two different conformations that were mutually exclusive and statistically distributed. The refined ratio between these two alternative conformations is 0.639(7):0.361(7). Crystal data are reported in Table S4. CCDC 2333985 contains supplementary crystallographic data for the compound under study. These data are provided free of charge by the joint Cambridge Crystallographic Data Centre and Fachinformationszentrum Karlsruhe Access Structures service.

## 4. Conclusions

Differently decorated chiral 1,3-dithiolanes represent a heterocyclic scaffold that is present in several promising compounds with pharmacological potential. Despite their importance, to the best of our knowledge, the resolution of these compounds is challenging and not routinary, and they are frequently synthesized and tested as racemates.

By applying complementary approaches, in this paper, we tackled the resolution of the versatile synthetic building block (1,4-dithiaspiro[4.5]decan-2-yl)methanol (*R/S*)-**1**. Our initial attempts at preparing **1** enantiomers encountered several challenges, including limited separability of diastereomeric pairs derived from the racemic alcohol and insufficient enantioselectivity in the enzyme-catalyzed hydrolyses of the corresponding ester derivatives. Enantioselective HPLC separation emerged as the most suitable method, offering both efficiency and speed for a further preparative scale-up and allowing us to obtain both (+)-**1** and (-)-**1** enantiomers in proper amounts for further studies and AC assignment. Due to the unsuccessful crystallization attempts of the enantiomeric alcohol **1**, the AC was indirectly assigned by the synthesis of the single enantiomer (+)-**BS148**, starting from (+)-1. The single crystal of (+)-**BS148** was subjected to X-ray diffraction analysis, enabling the attribution of the (*R*)- AC. Thus, by means of an indirect correlation, we assigned the AC (*R*)-(+)-**1** and (*S*)-(−)-**1** to the enantiomers of the target alcohol.

The enantiomers of **1** and **BS148** represent a viable reference standard for determining the AC of chiral 1,3-dithiolane derivatives by chemical correlation or comparison of the ECD or VCD spectra and elution order on CSPs.

Moreover, the enantioselective high-performance liquid chromatography proposed herein for the resolution of (*R/S*)-**1** may be scaled up further, thus providing a practical method for the preparation of this chiral scaffold to researchers involved in the synthesis of 1,3-dithiolane-based pharmaceutically relevant compounds.

## Figures and Tables

**Table 1:** Enzymes tested in this work.

Name	Enzyme	Immobilization Carrier
Acylase	*Aspergillus melleus* acylase	Relizyme 112/S
ANL	*Aspergillus niger* lipase fraction	OC-AG
ANE	*Aspergillus niger* esterase fraction	Relizyme 112/S
AXE	*Bacillus pumilus* esterase	GLX-AG
CRL	*Candida rugosa* lipase	OC-AG
Novozym435^®^	*Candida antarctica* lipase	Acrylic resin
PCL	*Pseudomonas cepacia* lipase	OC-AG
PFL	*Pseudomonas fluorescens* lipase	OC-AG
Protease N	*Bacillus subtilis* protease	GLX-AG
PSL	*Pseudomonas stutzeri* lipase	OC-AG
TLL	*Thermomyces lanuginosus* lipase	OC-AG

OC-AG = octyl-agarose; GLX-AG = glyoxyl-agarose; Relizyme 112/S = epoxy-derivatized methacrylic carrier.

## Data Availability

The original crystallographic data presented in the study are openly available in the Cambridge Crystallographic Data Centre at CCDC 2333985. All other data are contained within the article or Supplementary Materials.

## Supplementary Materials

The following supporting information can be downloaded at: https://www.mdpi.com/article/10.3390/ijms252312880/s1, References [32,33,34] are cited in the Supplementary Materials.
