# Supplementary material for: 1,3-Dithiolane as a Privileged Scaffold in Bioactive Derivatives: Chiral Resolution and Assignment of Absolute Configuration
Source: Int J Mol Sci. 2024 Nov 29;25(23):12880. doi: 10.3390/ijms252312880 (PMC11641153; doi:10.3390/ijms252312880)
Supplement: Supplementary file 1 [file ijms-25-12880-s001.zip › ijms-3313066-supplementary.pdf]

Supporting Information
1,3-Dithiolane as a Privileged Scaffold in Bioactive Derivatives: Chiral Resolution and Assignment of Absolute Configuration

Roberta Listro[1], Giacomo Rossino[1], Valeria Cavalloro[2], Daniela Rossi[1], Massimo Boiocchi[3], Marina Simona Robescu[1], Teodora Bavaro[1], Silvia Franchini[4], Claudia Sorbi[4], Marco De Amici[5], Pasquale Linciano[1] and Simona Collina[1*]

1 *Department of Drug Sciences, University of Pavia, Viale Taramelli 12, 27100 Pavia, Italy; roberta.listro@unipv.it Giacomo.rossino@unipv.it Daniela.rossi@unipv.it marinasimona.robescu@unipv.it teodora.bavaro@unipv.it pasquale.linciano@unipv.it simona.collina@unipv.it*
2 *Department of Earth and Environmental Sciences, University of Pavia, Via Sant'Epifanio 14, 27100 Pavia, Italy; valeria.cavalloro@unipv.it*
3 *Centro Grandi Strumenti, University of Pavia, Via Bassi 21, 27100 Pavia, Italy; massimo.boiocchi@unipv.it*
4 *Department of Life Sciences, University of Modena and Reggio Emilia, Via Campi 103, 41125 Modena, Italy; silvia.franchini@unimore.it claudia.sorbi@unimore.it*
5 *Department of Pharmaceutical Sciences, University of Milan, Via Luigi Mangiagalli 25, 20133 Milan, Italy; marco.deamici@unimi.it*
* *Correspondence: simona.collina@unipv.it*

General procedure for diastereomer derivatization of alcohol 1

To a solution of DCC (1 equiv.) and (±)-**1** (1 equiv.) in anhydrous DCM at 0 °C under nitrogen atmosphere, a solution of either L-amino acid or (S)-(+)-10-camphorsulfonyl chloride (1 equiv.) in anhydrous DCM was added. The reaction mixture was stirred under the same conditions until complete consumption of starting materials (monitored by TLC using n-hexane/EtOAc, 3:2 v/v as mobile phase). The reaction was quenched with EtOAc, and the organic phase was washed with saturated Na_2CO_3 solution, dried over anhydrous Na_2SO_4, and concentrated. The crude product was purified by flash chromatography using the following solvent systems: for Fmoc-L-phenylalanine derivative: n-hexane/EtOAc (4:1, v/v); for (S)-(+)-10-camphorsulfonyl chloride derivative: n-hexane/EtOAc (8:2, v/v); for (R)-(+)-3,4-dimethyl ariloxipropionic acid derivative: n-hexane/EtOAc (8:2, v/v); for Fmoc-R-tyrosine(tert-butyl)-OH derivative: n-hexane/EtOAc (7:3, v/v). No separation of diastereomers was achieved.

Enzymatic approach

General information

Octyl Sepharose® CL-4B carrier, tripropionine, 1-naphtyl acetate and the crude extracts of lipases from Aspergillus niger (ANL), *Candida rugosa* (CRL), *Pseudomonas cepacia* (PCL), *Pseudomonas fluorescens* (PFL), and *Thermomyces lanuginosus* (TLL) were

purchased from Sigma-Aldrich (Milano, Italy). Acetyl xylan esterase (AXE) from *Bacillus pumilus* was a kind gift from Acs Dobfar (Tribiano, Italy). Commercially available lipase from *Pseudomonas stutzeri (PSL*) was purchased from Meyto Sangyo (Japan).

The crude extracts of protease N from *Bacillus subtilis* and acylase *Aspergillus melleus* were kindly donated by Amano Enzyme Europe (Chipping Norton, UK). Novozym435® was a kind gift of Novozymes (Bagsvaerd, Denmark).

Sepharose™ CL-6B (agarose) carrier was from GE Healthcare (Milan, Italy). ReliZyme 112/S was a kind gift of Resindion (Binasco, Milano, Italy).

Determination of enzymatic activity

The activity of the enzymes was determined following a standard protocol by using an automatic titrator pH-Stat. The hydrolytic activity was calculated based on NaOH consumption (mL of NaOH/min).

a) Standard activity assay of lipases

The activity of lipases was determined using tripropionin as standard substrate[1]. The standard reaction mixture was composed of 0.6 mL of acetonitrile, 1 mL of tripropionin, and 18.4 mL of Tris-HCl (25 mM, pH 7.0). The reaction was started through the addition of a soluble enzyme opportunely diluted or immobilized enzyme (10–15 mg). The mixture was mechanically stirred, and pH was maintained at 7.0 using 100 mM NaOH as titrant. Experiments were done at least in duplicate.

b) Standard activity assay of esterases

The activity of esterases was determined using 1-naphthyl acetate as a standard substrate[1]. The standard reaction mixture was composed of 2 mL of acetonitrile, 2 mL of 1-naphthyl acetate (50 mM in acetonitrile), and 16 mL of phosphate buffer (25 mM, pH 7.0). The reaction was started through the addition of a soluble enzyme opportunely diluted or immobilized enzyme (10–15 mg). The mixture was mechanically stirred, and pH was maintained at 7.0 using 100 mM NaOH as titrant. Experiments were done at least in duplicate.

Enzyme immobilization

For all the immobilization procedures, a 10:1 ratio volume of immobilization reaction/volume of the carrier was used. During immobilization the residual activity of the supernatant (100 μL) as well as the activity of the final immobilized derivatives were checked by the standard activity assays described before.

Immobilization of lipases on octyl-Sepharose® (OC-AG) by adsorption

Lipases were immobilized on OC-AG as reported in Scheme SI-1[1]. Briefly, the crude extracts (1 g for freeze-dried preparations (except for ANL for which 2 g were

used) or 1 mL for liquid preparations) were suspended in KH_2PO_4 buffer (25 mM, pH 7.0). The mixture was allowed to stir on a rolling shaker for 30 minutes to obtain a homogeneous solution. Then, octyl-Sepharose® (1 g), previously conditioned with the same buffer, was added and the suspension was allowed to stir overnight at room temperature. The enzyme derivatives were filtered under reduced pressure on a Büchner funnel, rinsed thoroughly with distilled water and stored at 4 °C till use. The immobilization yield (%) in terms of activity and the final activity of each immobilized derivative are reported in Table SI-1.

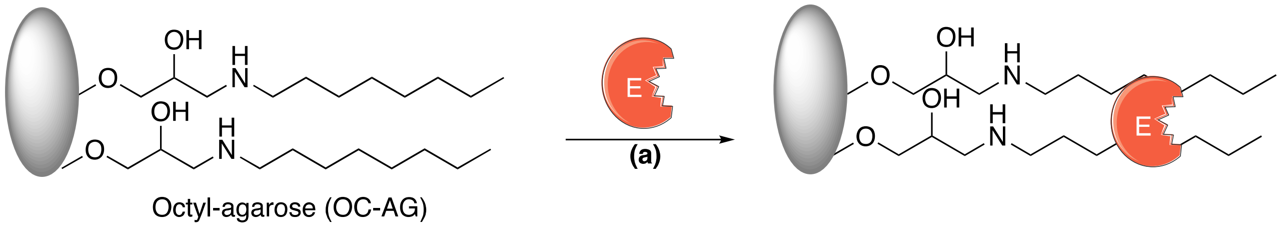

Figure S1. (a) 25 mM KH_2PO_4 pH 7.0, RT, 18 h, mechanical stirring. Loading 1 g of crude extract (except for ANL for which 2 g were used) or 1 mL of liquid preparation per 1 g of carrier.

Table S1. Immobilization yield and derivative activity. (a) The immobilization yield was calculated as follows: immobilized activity (U)/initial activity (U) x 100; (b) Novozym435® commercial preparation: CALB immobilized on acrylic resin.

Enzyme	Source	Immobilization yield (%)[(a)]	Derivative activity (U/g)
CALB[(b)]	*Candida antarctica*	-	773
ANL	*Aspergillus niger*	100	94
CRL	*Candida rugosa*	90	800
PCL	*Pseudomonas cepacia*	98	694
PFL	*Pseudomonas fluorescens*	94	1353
PSL	*Pseudomonas stutzeri*	100	790
TLL	*Thermomyces lanuginosus*	99	1000

<u>*Immobilization of AXE and Protease N on glyoxyl-agarose (GLX-AG) by covalent binding*</u>

GLX-AG was prepared as reported in literature[2]. Briefly, Sepharose™ CL-6B (agarose, 5 g) was suspended in deionized H_2O (1.4 mL) and NaOH (1.7 M, 2.4 mL) containing $NaBH_4$ (28.4 mg/mL). Subsequently, glycidol (1.7 mL) was added dropwise keeping the vessel at 4 °C in an ice bath. The reaction was kept under gently stirring overnight at 25 °C. After the incubation period, the suspension was filtered and the carrier was washed abundantly with deionized H_2O. Oxidation was initiated by adding $NaIO_4$ (100 mM, 34 mL). The reaction was carried out for 2 h at room temperature, then the carrier was filtered under reduced pressure and washed abundantly with deionized H_2O and stored at 4 °C.

Immobilization of AXE and Protease N on GLX-AG was performed as depicted in Figure SI-2 following standard protocols[3]. Briefly, glyoxyl-agarose was washed

abundantly with $NaHCO_3$ buffer (50 mM, pH 10) and then filtered under reduced pressure until dryness. The enzymes (150 mg of AXE (49 mg/mL) and 1 g of crude extract of Protease N) were solubilized into $NaHCO_3$ buffer (50 mM, pH 10). Then, the carrier (1 g) was added and the suspension was allowed to stir for 18 h at 4 °C for AXE and for 3 h at 25 °C for Protease N, respectively. Finally, $NaBH_4$ (1 mg for each 100 mg of carrier for AXE and 1 mg for each mL of immobilization for Protease N) was added to the mixture and incubated for 30 minutes at 4 °C for AXE and at 25 °C for Protease N, respectively, to allow imino bonds reduction. The immobilized enzymes were then filtered, rinsed thoroughly with distilled water and stored at 4 °C till use. The immobilization yield (%) in terms of activity and the final activity of AXE immobilized derivative are reported in Table SI-2. For Protease N the standard substrate for the activity assay was not available in house thus the immobilization was not monitored as well as the final activity of the derivative was not tested.

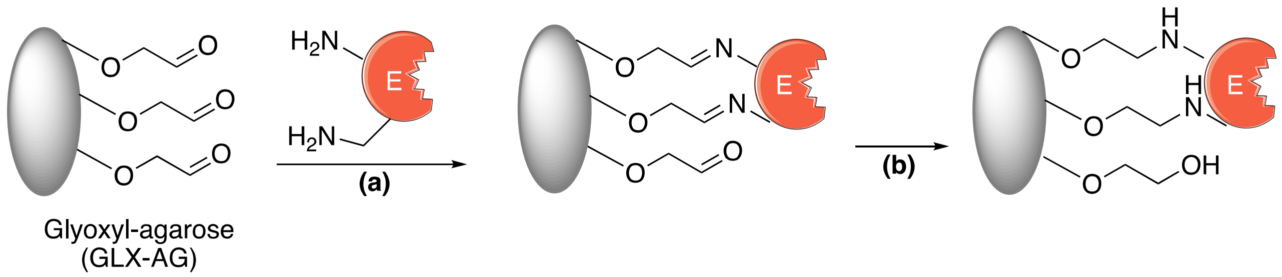

Figure S2. (a) 50 mM $NaHCO_3$ pH 10, 4°C and 18 h for AXE and RT and 3 h for Protease N, mechanical stirring. Loading 150 mg of AXE and 1 g of crude extract for Protease N per 1 g of carrier. (b) 1 mg/100 mg carrier for AXE or 1 mg/mL for Protease N of $NaBH_4$, 4 °C for AXE and RT for Protease N, 30 min, mechanical stirring.

Table S2. Immobilization yield and derivative activity. (a) The immobilization yield was calculated as follows: immobilized activity (U)/initial activity (U) x 100.

Enzyme	Source	Immobilization yield (%)(a)	Derivative activity (U/g)
AXE	Acetyl xylan esterase from *Bacillus pumilus*	48	280

<u>*Immobilization of ANE and Acylase on ReliZyme 112/S by covalent binding*</u>

The esterase fraction of ANL preparation and Acylase from *Aspergillus melleus* were immobilized on ReliZyme 112/S as depicted in Figure SI-3 following standard protocols [4,5].

The supernatant of ANL after being submitted to immobilization of the lipase fraction on OC-AG (as described previously), was enriched in an esterase activity fraction. This supernatant was diluted 1:1 with a solution of 2 M phosphate buffer at pH 7.5. ReliZyme 112/S (1 g), previously washed with the same buffer, was added

and the suspension was stirred at room temperature for 24 h. The enzyme derivative was then filtered, washed with distilled water and stored at 4 °C till use[4].
The crude extract of Acylase (1 g) was suspended in potassium phosphate buffer 1 M pH 8.0 (14 mL). Then, the carrier (1 g) was added, and the suspension was allowed to stir for 24 h at room temperature. The immobilized enzyme was then filtered, rinsed thoroughly with distilled water and stored at 4 °C till use[5]. The immobilization yield (%) in terms of activity and the final activity of immobilized derivatives are reported in Table SI-3.

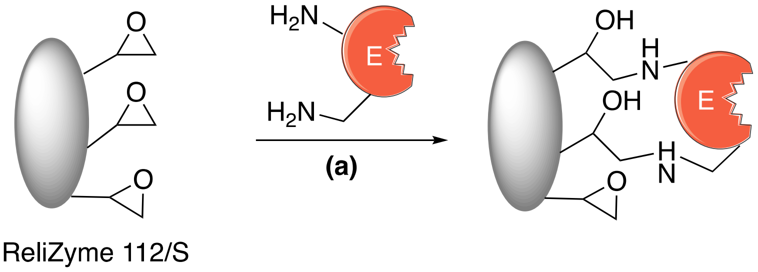

Figure S3. (a) 1 M KH_2PO_4 pH 8, RT, 24 h, mechanical stirring. Loading 2 g of crude extract after OC-GLX immobilization for ANE and 1 g of crude extract for 1 g carrier for Acylase.

Table S3. Immobilization yield and derivative activity. (a) The immobilization yield was calculated as follows: immobilized activity (U)/initial activity (U) x 100.

Enzyme	Source	Immobilization yield (%)(a)	Derivative activity (U/g)
ANE	*Aspergillus niger*	33	12
Acylase	*Aspergillus melleus*	83	7.3

Biocatalyzed reaction procedure and monitoring

Compound (*R*/*S*)-**2** was dissolved in a mixture of *tert*-butanol and water (90:10 v/v), thus obtaining a concentration of 2 mM (for the initial preliminary screening) to 10 mM (for subsequent investigations). Before adding the enzyme, a sample was collected at t0 and subjected to TLC analysis (*n*-Hex:EtOAc, 8:2, v/v, ceric sulfate/ammonium molybdate solution detection). 68 Enzymatic units per mg of substrate (*R*/*S*)-**2** were added, and the reactions were incubated at room temperature on a rolling shaker. Periodic samples were withdrawn for TLC (alcohol R_f: 0.40) and chiral HPLC analysis (50-250 µL were dried with N_2 and solubilized in 100µL of HPLC mobile phase).

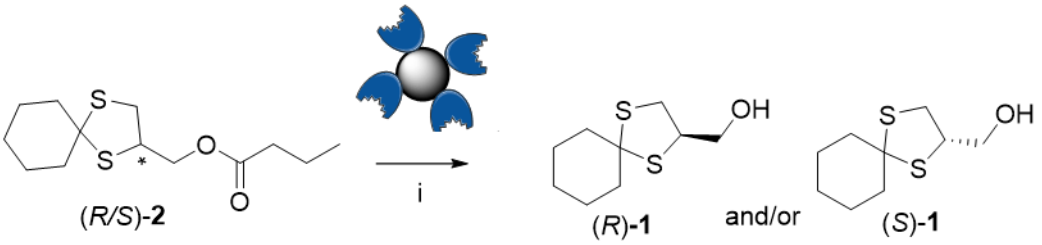

Scheme S1. Hydrolysis of (*R*/*S*)-**2** under the catalysis of immobilized enzymes listed in Table 1. (i) Reaction conditions: 90 % *tert*-butanol and 10 % H_2O (9 mL), concentration range of ester substrate 2-10 mM (5-10 mg), 68 UI per mg of substrate, r. t., 24 h, rolling shaking.

Screening of chiral stationary phase and mobile phase

Table S4. Results of the screening protocol for alcohol **1**, expressed in terms of retention factor (k_1 and k_2), separation factor (α), and resolution factor (Rs). Eluent composition: A, n-Hex/ IPA (90:10 v/v); B, n-Hex/IPA (80:20 v/v); C, IPA (100); D, EtOH (100); E, n-Hex/EtOH (80:20 v/v); F, n-Hex/EtOH (90:10 v/v); G, n-Hex/MTBE (90:10 v/v); H, n-Hex/MTBE (80:20 v/v); Flow rate: 1 mL/min [a]Flow rate: 0.5 mL/min; Concentration: 1 mg/mL; Injection volume: 10 µL; UV detection at 220 nm.

CSPs								
	Chiralpak IA				*Chiralpak IC*			
Eluent	k_1	k_2	α	*RS*	k_1	k_2	α	*RS*
A	1.21	1.33	1.09	1.12	0.67	-	-	-
B	0.57	0.63	1.10	0.65	0.36	-	-	-
C[a]	0.69	-	-	-	0.19	-	-	-
D	1.51	1.64	1.09	0.83	0.18	-	-	-
E	1.10	1.31	1.19	1.50	0.32	-	-	-
F	1.18	1.31	1.11	1.13	0.69	-	-	-
G	Over 80 min		-	-	Over 80 min		-	-
H	4.60	5.34	1.16	1.39	3.41	-	-	-

Example of the analytical screening of enzymatic hydrolysis

The monitoring chromatograms of ANE hydrolysis have been reported.

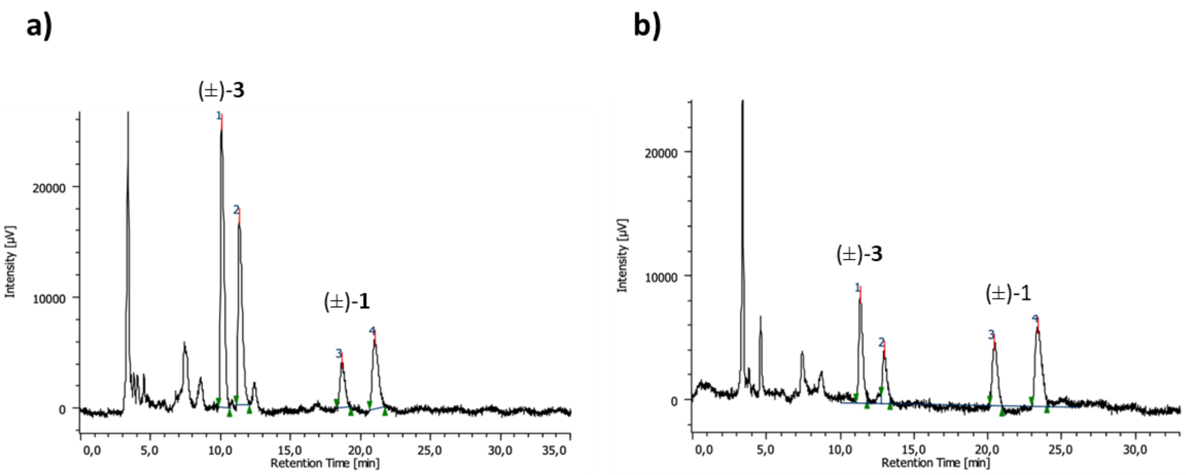

a)

(±)-3		(±)-1	
I eluted	*II eluted*	*I eluted*	*II eluted*
Tr=10.05	Tr=11.32	Tr=18.61	Tr=20.98
A%=40.9	A%=32.8	A%=9.26	A%=17.2
$\alpha_{1\text{-}2}$=1.19		$\alpha_{3\text{-}4}$=1.16	
$RS_{1\text{-}2}$= 2.57		$RS_{3\text{-}4}$= 3.38	

b)

(±)-3		(±)-1	
I eluted	*II eluted*	*I eluted*	*II eluted*
Tr=11.36	Tr=12.94	Tr=20.39	Tr=23.37
A%=27.9	A%=15.2	A%=21.7	A%=35.3
$\alpha_{1\text{-}2}$=1.20		$\alpha_{3\text{-}4}$=1.18	
$RS_{1\text{-}2}$= 3.19		$RS_{3\text{-}4}$= 3.90	

Figure S4. HPLC monitoring of two hydrolysis samples with ANE enzyme picked up after 6 h (**a**) and 24 h (**b**). Chromatographic condition: Chiralpak IA (4,6mm I.D. x 250 mm), Mobile phase: n- Hex/MTBE 4:1 (v/v), Flow rate:1 mL/min, 25°C, λ=220 nm. Injection volume: 10 µL.

The chromatograms of the two different time points using acylase enzyme have been reported.

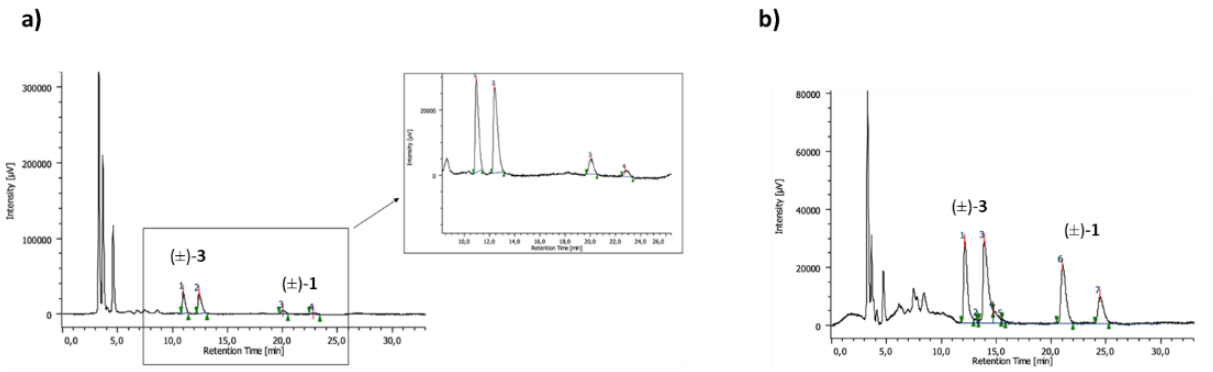

a)

(±)-3		(±)-1	
I eluted	*II eluted*	*I eluted*	*II eluted*
Tr=10.94	Tr=12.38	Tr=21.10	Tr=24.39
A%=39.7	A%=47.2	A%=9.1	A%=3.9
$\alpha_{1\text{-}2}$=1.19		$\alpha_{3\text{-}4}$=1.16	
$RS_{1\text{-}2}$= 2.68		$RS_{3\text{-}4}$= 3.80	

b)

(±)-3		(±)-1	
I eluted	*II eluted*	*I eluted*	*II eluted*
Tr=12.11	Tr=13.88	Tr=21.10	Tr=24.39
A%=24.7	A%=33.8	A%=23.5	A%=12.4
$\alpha_{1\text{-}2}$=1.21		$\alpha_{3\text{-}4}$=1.16	
$RS_{1\text{-}2}$= 2.57		$RS_{3\text{-}4}$= 3.38	

Figure S5. HPLC monitoring of two hydrolyzed samples with Acylase enzyme picked up after 6 h (**a**) and 24 h (**b**). Chromatographic condition: Chiralpak IA (4,6mm I.D. x 250 mm), Mobile phase: n- Hex/MTBE 4:1 (v/v), Flow rate:1 mL/min, 25°C, λ=220 nm. Injection volume: 10 μL.

X-ray crystallographic study

Crystal data obtained for (+)-**BS148** by X-ray diffraction analysis are reported.

Table S5. Crystal data of (+)-**BS148**

Formula	$C_{21}H_{31}NS_2$	scan type	ω scans
M	361.59	θ range (°)	2.31 - 25.03
Dimension (mm)	0.62 x 0.54 x 0.42	measured reflections	9344
crystal system	orthorhombic	unique reflections	3598
space group	*P* $2_12_12_1$ (no. 19)	R_{int}	0.032
a (Å)	6.402(2)	strong data [$I_O>2\sigma(I_O)$]	2544
b (Å)	14.039(2)	refined parameters	290
c (Å)	22.691(4)	*R*1, *wR*2 strong data	0.0536, 0.0791
V (Å^3)	2039.4(7)	*R*1 *wR*2 all data	0.0872, 0.0940
Z	4	GoF	1.171
ρ_{calcd} (g cm^{-3})	1.178	Flack x parameter	-0.01(7)
μ Mo-Kα [mm^{-1}]	0.264	max/min residuals (eÅ^{-3})	0.13/-0.11

References

1. Tanzi, L.; Robescu, M.S.; Marzatico, S.; Recca, T.; Zhang, Y.; Terreni, M.; Bavaro, T. Developing a Library of Mannose-Based Mono- and Disaccharides: A General Chemoenzymatic Approach to Monohydroxylated Building Blocks. *Molecules* **2020**, *25*, 5764, doi:10.3390/molecules25235764.
2. Bruni, M.; Robescu, M.S.; Ubiali, D.; Marrubini, G.; Vanna, R.; Morasso, C.; Benucci, I.; Speranza, G.; Bavaro, T. Immobilization of γ-Glutamyl Transpeptidase from Equine Kidney for the Synthesis of Kokumi Compounds. *ChemCatChem* **2020**, *12*, 210–218, doi:10.1002/cctc.201901464.
3. Robescu, M.S.; Annunziata, F.; Somma, V.; Calvio, C.; Morelli, C.F.; Speranza, G.; Tamborini, L.; Ubiali, D.; Pinto, A.; Bavaro, T. From Batch to Continuous Flow Bioprocessing: Use of an Immobilized γ-Glutamyl Transferase from B. Subtilis for the Synthesis of Biologically Active Peptide Derivatives. *J. Agric. Food Chem.* **2022**, *70*, 13692–13699, doi:10.1021/acs.jafc.2c03702.
4. Bavaro, T.; Torres-Salas, P.; Antonioli, N.; Morelli, C.F.; Speranza, G.; Terreni, M. Regioselective Deacetylation of Disaccharides via Immobilized Aspergillus Niger Esterase(s)-Catalyzed Hydrolysis in Aqueous and Non-Aqueous Media. *ChemCatChem* **2013**, *5*, 2925–2931, doi:10.1002/cctc.201300388.

5. Bavaro, T.; Cattaneo, G.; Serra, I.; Benucci, I.; Pregnolato, M.; Terreni, M. Immobilization of Neutral Protease from Bacillus Subtilis for Regioselective Hydrolysis of Acetylated Nucleosides: Application to Capecitabine Synthesis. *Molecules* **2016**, *21*, 1621, doi:10.3390/molecules21121621.
